# TREK-1 in the heart: Potential physiological and pathophysiological roles

**DOI:** 10.3389/fphys.2022.1095102

**Published:** 2022-12-22

**Authors:** Emilie Bechard, Jamie Bride, Jean-Yves Le Guennec, Fabien Brette, Marie Demion

**Affiliations:** PhyMedExp, Université de Montpellier, Inserm U1046, UMR CNRS 9412, Montpellier, France

**Keywords:** TREK-1, K2P2.1, atrial fibrillation, myocardial infarction, RVOT

## Abstract

The TREK-1 channel belongs to the TREK subfamily of two-pore domains channels that are activated by stretch and polyunsaturated fatty acids and inactivated by Protein Kinase A phosphorylation. The activation of this potassium channel must induce a hyperpolarization of the resting membrane potential and a shortening of the action potential duration in neurons and cardiac cells, two phenomena being beneficial for these tissues in pathological situations like ischemia-reperfusion. Surprisingly, the physiological role of TREK-1 in cardiac function has never been thoroughly investigated, very likely because of the lack of a specific inhibitor. However, possible roles have been unraveled in pathological situations such as atrial fibrillation worsened by heart failure, right ventricular outflow tract tachycardia or pulmonary arterial hypertension. The inhomogeneous distribution of TREK-1 channel within the heart reinforces the idea that this stretch-activated potassium channel might play a role in cardiac areas where the mechanical constraints are important and need a particular protection afforded by TREK-1. Consequently, the main purpose of this mini review is to discuss the possible role played by TREK -1 in physiological and pathophysiological conditions and its potential role in mechano-electrical feedback. Improved understanding of the role of TREK-1 in the heart may help the development of promising treatments for challenging cardiac diseases.

## 1 Introduction

In cardiomyocytes, excitation-contraction coupling, the process that links electrical activity to cell contraction, is well established. On the contrary, the mechano-electric feedback (MEF) mechanism that can be summarized by how mechanical stimulation can modulate action potential (AP) shape is less understood. Such a feed-back mechanism must involve mechanosensitive signals, mainly stretch, especially *via* mechano-gated ionic channels.

K2P channels are modulators of cardiac repolarization and within this family of channels, TREK-1 is the most abundant channel expressed in the human heart (Wiedmann et al., 2021). The mechano-gated TREK-1 channel is differentially expressed in regions of the heart participating in electrical heterogeneity within specific areas (Tan et al., 2004). TREK-1 is 17 time more express at the mRNA level in the endocardium when compared to epicardium, and 3-fold at the functional (current) level (Kelly et al., 2006).

## 2 TREK-1 and mechano-electrical feedback

As well as TREK-1 channel expression is heterogenous in ventricle, MEF differs regionally. In 1999, it was demonstrated that, in response to stretch, canine AP was shortened at the endocardial level but not in the epicardium leading to ventricular arrhythmias (Takagi et al., 1999). Such stretch-induced arrhythmias were sensitive to gadolinium, a non-specific stretch-activated channel blocker. In 2006, Kelly and collaborators demonstrated that in Langendorff perfused rat isolated heart, increasing intraventricular pressure by inflation of a balloon, induced a shortening of the AP both in subepicardial and subendocardial zones (Kelly et al., 2006). However, AP shortening was more pronounced in the endocardium than in the epicardium at all repolarization levels. This suggests that the spatial variation of TREK-1 channel expression may influence regionally the MEF altering in turn the dispersion of AP repolarization and induces disturbances in repolarization in several models including human (Sarubbi et al., 2004). Such repolarization dispersion (*i.e.* AP at different part of the ventricle repolarizing at different times) is arrhythmogenic (Roden and Woosley, 1985) and is normally reduced under physiological conditions. The different AP durations (APD) in the myocardium finally synchronize their repolarization along the wall despite an asynchronous depolarization time (Volders et al., 2000).

However, in several cardiac pathologies, both TREK-1 channel expression and MEF are modified. In pathological conditions where mechanical properties are altered, such as fibrosis, a slowed AP propagation may induce a dispersion of repolarization and thus arrhythmias, altering both MEF and APD regulation.

For example, in rat, TREK-1 channel expression is increased by cardiac hypertrophy induced by isoproterenol and this is associated with an amplified transmural gradient (endocardium vs*.* epicardium) of TREK-1 channel (Wang et al., 2013). This up-regulation might be linked to reexpression of fetal genes, characteristic finding of pressure overload–induced cardiac hypertrophy, however to the best of our knowledge there is no information about a possible developmentally regulated expression of TREK-1 gene. In mammals, global deletion of TREK-1 induces an exaggerated concentric hypertrophy in response to pressure overload, but protects from the development of systolic and diastolic dysfunction. As TREK-1 is present in cardiac fibroblasts and modulates their function, its specific deletion in fibroblasts reduces fibrosis development. Thus, cardioprotective effects of TREK-1 loss of function are driven by cardiac fibroblasts (Abraham et al., 2018).

TREK-1 activity can also be changed during pathology since the activation of TREK-1 by Polyunsaturated fatty acids like arachidonic acid might occur in pathophysiological situations like ischemia when ATP cause the release of this fatty acid (Aimond et al., 2000).

However, the transmural variation of TREK-1 expression cannot explain by itself all the MEF variability. Indeed, the cellular strain and forces are locally different from the endocardial to epicardial cells when the ventricle is deformed. This was first shown at the cellular level (Cazorla et al., 2000) and more recently in living individuals cardiac layers (Pitoulis et al., 2020). For example, during end-diastolic relaxation, significant circumferential stretch, wall thinning, and in-plane and transverse shear were observed. Left ventricular mechanics during the early relaxation phase involves substantial deformation of fiber and sheet structures with significant transmural heterogeneity. A significant epicardial stretch along myofibers was observed during early relaxation when compared to endocardial fibers (Ashikaga et al., 2004).

## 3 Physiological role of TREK-1

While some changes in the expression of TREK-1 might appear in pathological circumstances, a putative physiological role of TREK-1 has never been shown. Knowing its electrophysiological properties, channel open at rest and outwardly rectifying current, led to the inference that this channel must hyperpolarize the resting membrane potential and/or shorten the APD of cardiac cells but this has never been shown (Benoist et al., 2014; Kelly et al., 2006). The reason for this is probably that there is no specific pharmacology for TREK-1 and that TREK-1 blockers are often other potassium conductance blockers (Wiedmann et al., 2021). It has to be underlined that the co-expression of K_IR_2.1, the molecular identity responsible for the current responsible of the cardiac resting membrane potential I_K1_, and TREK-1 shows that TREK-1 influences slightly the resting membrane potential (Decher et al., 2017b). Also, there are many cases of mutations in channels carrying the rapid and slow delayed-rectifier and basal inward-rectifier potassium currents (I_Kr_, I_Ks_ and I_K1_, respectively), involved in the reserve of repolarization, that lead to long QT syndrome (Crotti et al., 2020). To the best of our knowledge, such mutations have never been described for TREK-1. This does not mean that I_TREK-1_ is not involved in ventricular repolarization, since such mutations can be lethal or induce other kind of adaptation.

Interestingly, it has been observed that TREK-1 channel is mainly expressed in the more mechanical constrained area of the heart. For example, TREK-1 is more abundant in endocardial cells than in epicardial cells (Tan et al., 2004; Kelly et al., 2006). Hence, the effects on AP shortening of inflating a balloon in the left ventricle are more pronounced in the endocardium than in the epicardium. Besides these observations, endocardial cells are known to be more rigid than epicardial cells (Cazorla et al., 1997, 1999). In addition, Gannier et al. (1994), observed that stretching single cells can induce a large increase in diastolic calcium. This phenomenon can occur for small sarcomere lengthening that is coherent with stiff endocardial cells. This phenomenon is accompanied by a stable membrane depolarization that is at the origin of the diastolic calcium increase (White et al., 1993; Gannier et al., 1996). Thus, this phenomenon, linked to the presence of inward stretch-activated current, must be prevented by the presence of TREK-1 that would compensate the inward current, especially in cells more prone to present this phenomenon, i.e., stiffer cells, endocardial cells.

The right ventricular outflow tract (RVOT) is also a region of the heart with high physical strain. This might explain, in part at least, that the RVOT is a region of the heart where arrhythmic syndromes and the majority of idiopathic ventricular arrhythmias are found in the absence of any structural modifications and without any known etiology (Srivathsan et al., 2005). It has been noted that in rat ventricle, the RVOT APD was shorter than in the other part of the right ventricle even though there was no evidence that it was due to an overexpression or overactivity of TREK-1 (Benoist et al., 2014).

However, there are some evidences of the role of TREK-1 remodeling in the development of some arrhythmias (Benoist et al., 2014; Kamatham et al., 2019).

## 4 TREK-1 and pathological condition

While the physiological role of TREK-1 has not been proven formally, some cardiac diseases have been shown to be associated with changes in TREK-1 expression and the implication of TREK-1 can be inferred based on its electrophysiological properties or even proved by restoration of the initial expression (Zhao et al., 2011; Benoist et al., 2014; Lugenbiel et al., 2017; Kamatham et al., 2019; Dragasis et al., 2022).

### 4.1 RVOT

The RVOT is the area of the right ventricle through which blood flows in to be expulsed in the pulmonary artery. The RVOT is under a high physical strain and is known to express TREK-1. A significant proportion of ventricular extrasystoles originate from the outflow tracts, left or right (Dragasis et al., 2022).

There are at least two kinds of tachycardia that have the RVOT as the locus for arrhythmias.

#### 4.1.1 Mutated leaky TREK-1 channel

RVOT ventricular tachycardia (RVOT-TC) is a common form of monomorphic ventricular tachycardia without any structural disease and without any explanation. 10% of ventricular tachycardia are idiopathic and interestingly 70% of these arrhythmias originate in the RVOT (Goldstein, 2017). While they are considered as benign, it has been shown that they can degenerate in ventricular fibrillation, often preceded by episodes of syncope (Noda et al., 2005). Decher et al. (2017b) identified a patient with a point mutation in TREK-1, I267T, located directly before the selectivity filter of the second pore loop. This mutation, that might be dominant-negative, makes TREK-1 permeable to sodium. Also, TREK-1 has an increased sensitivity to stretch accompanied by a reduced desensitization of the channel. Episodes of RVOT-TC are generally observed in situations where the sympathetic system is activated and thus normally TREK-1 is more or less inhibited. Decher et al. (2017b) showed that the activation of ß_1_-receptors, co-expressed in oocytes with TREK-1^I267T^, induces a larger depolarizing inward sodium current and thus a larger depolarization that must participate as such to the development of arrhythmias. Indeed, the increased entry of sodium leads to an increased entry of calcium through the sodium-calcium exchanger that in turn produces arrhythmias due to the transient inward current I_TI_ (Lederer and Tsien, 1976). It has to be noted that another gene modification, an alternative translation initiation, leads to an increased sodium permeability (Thomas et al., 2008). However, the normal and pathological expression of this variant is not known as well as the physiological sodium permeability (Decher et al., 2017b; 2017a). Interestingly, the I267T mutant can recover its potassium selectivity thanks to BL-1249, a TREK-1 channel activator, opening hopes in the treatment of such a rare disease.

#### 4.1.2 Pulmonary arterial hypertension

Pulmonary arterial hypertension (PAH) is a cardiac disease resulting from to a vasoconstriction and/or cell proliferation in the pulmonary circulation. The main consequences of PAH are right ventricular hypertrophy, arrhythmias and failure. A rat model of PAH has been developed by injecting monocrotaline, a plant alkaloid. Benoist et al. (2014) used that model to understand the evolution of remodeling associated with heart failure and the occurrence of arrhythmias. They found that while the APD in the RVOT is shorter than in the rest of the right ventricle in healthy animals, this APD increased more in the RVOT with PAH. The authors showed thus that remodeling is more important in the RVOT than in the other areas of the right ventricle and that such changes in APD in adjacent part of the ventricle can be responsible of arrhythmias due the dispersion of repolarization. While there was no functional analysis performed to explain such a lengthening of the AP, (Benoist et al., 2014), showed that the level of mRNA coding for TREK-1 decreased only in the right ventricle, even though there was no particular focus on the RVOT. By correlating this decrease in TREK-1 gene expression, and the associated decrease in TRPC1 and increase in TRPC6 expression (Benoist et al., 2014), to the channel conductance’s, they showed that the increase in APD can be explained.

These findings are of great interest since they were counter-intuitive knowing that an acute stretch induces an increase of the TREK-1 protein (Zhao et al., 2007). A possible explanation might be that initially the acute strain induces an increase of TREK-1 to compensate and, with time, there is the installation of a chronic strain that induces a decompensation linked to, at least, a remodeling of TREK-1 expression.

### 4.2 Atrial fibrillation

Atrial fibrillation (AF), the most common arrhythmia, is associated with both structural and electrical remodeling that cause disturbance in repolarization and conduction and contribute to arrhythmogenesis (Schmidt et al., 2011; Wakili et al., 2011; Dobrev et al., 2012). TREK-1 expression in human atrium displays a relevant level that suggests functional and potential therapeutic roles (Schmidt et al., 2017).

Indeed, a down-regulation of *TREK-1* mRNA expression and protein level in atrium were observed in patients with AF complicated by heart failure (Schmidt et al., 2013, 2017; Lugenbiel et al., 2017). Down-regulation of TREK-1 was similarly observed in a tachypacing pig model of AF with reduced left ventricular ejection fraction. This downregulation of TREK-1 expression is consistent with a prolonged APD and effective refractory periods (ERP) but also with an increased susceptibility to experience extrasystoles. The downregulation of *TREK-1* mRNA in neonate ventricular cardiomyocytes from rat under mechanical stretch suggests that dilation of atria observed in AF patients may directly contribute to TREK-1 remodeling (Lugenbiel et al., 2017). Then, restoring TREK-1 expression in pig with AF using gene therapy was followed by a recovery of sinus rhythm and prolongation of the ERP was attenuated (Lugenbiel et al., 2017). Moreover, overexpressing TREK-1 by gene therapy enhances TREK-1 current and results in APD shortening in mouse cardiomyocytes (Lugenbiel et al., 2017). TREK-1 channel downregulation is clearly involved in the development of AF and this suggests a potential therapeutic significance of TREK-1 gene therapy to manage AF complicated by heart failure.

### 4.3 Myocardial infarction

Post-myocardial infarct arrhythmias are a major risk factor of sudden cardiac death. After myocardial infarction (MI), an electrical remodeling occurs, increasing the electrical instability of the post-MI heart (Huang et al., 2001). In 2002, Barrabés et al. (2002) demonstrated that MEF can be enhanced in postinfarcted myocardium when compared with normal cardiac muscle, leading to mechano-induced ventricular arrhythmias and consequently ventricular fibrillation (Barrabés et al., 2002). Zhao et al. (2011) showed that TREK-1 channel remodeling occurs after MI in both endocardium and epicardium. TREK-1 expression decreased in the infarcted region while it is increased in the infarcted border region as a compensatory mechanism. This remodeling of TREK-1 expression in infarcted border region, positively correlated with outward TREK-1 current, could be responsible of the post-MI arrhythmogenesis by inducing a dispersion of effective refractory periods in adjacent cardiac areas. A more recent study, using TREK-1 invalidated model, demonstrated that following coronary artery ligation, the infarct size was increased in TREK-1 knock-out mice, associated with higher systolic dysfunction when compared to wild-type mice (Kamatham et al., 2019). This study suggests that TREK-1 expression could be protective during ventricular remodeling by regulating membrane potential and thus calcium homeostasis.

During the process of cardiac work after an infarction, the effects of mechanical stretch on myocardium in different regions may produce an altered electrical activity, inducing disturbances in repolarization and electrical instability that is a critical factor for arrhythmogenesis.

In conclusion, the ventricle preload and wall thickness modifications are able to alter the electrical signal induced by the MEF. This signal provides the trigger and/or the substrate to ventricular arrhythmias.

## 5 Future directions

**FIGURE 1:**
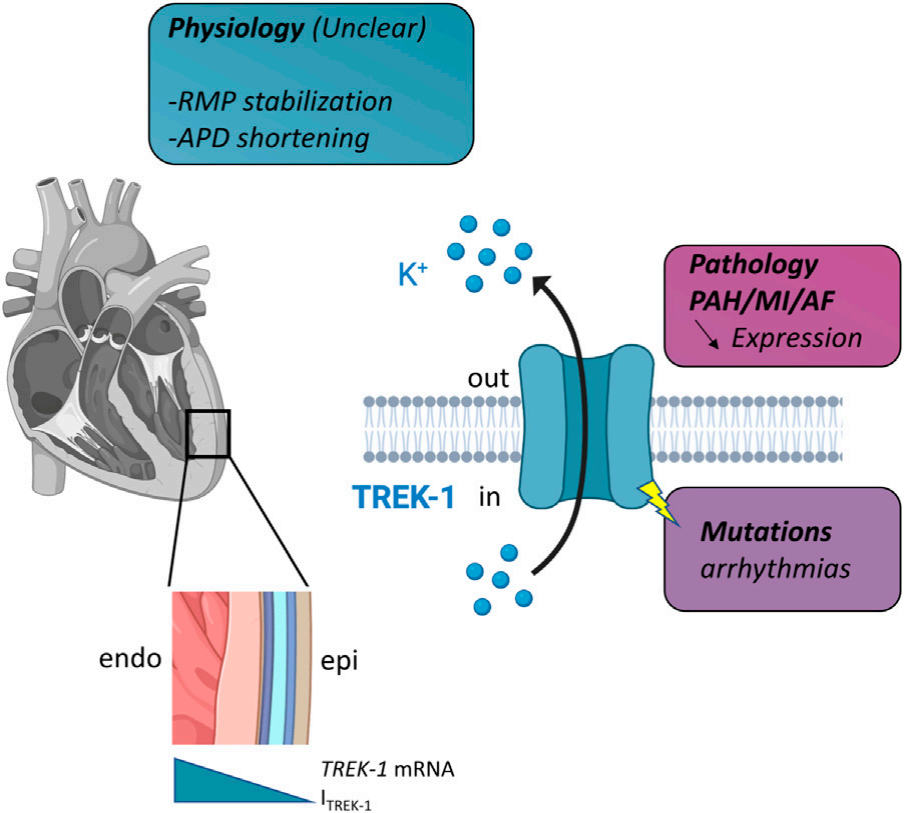
Schematic representation of TREK-1 involvement in cardiac physiology and pathology. Left, in the heart, there is a gradient of TREK-1 expression (hence current) between endocardium (endo) and epicardium (epi). Right, TREK-1 is a potassium channel and this physiological role is unclear. It may be involved in resting membrane potential (RMP) stabilization, action potential duration (APD) shortening. Decrease expression has been observed in several pathologies: pulmonary arterial hypertension (PAH), myocardial infarction (MI) and atrial fibrillation (AF). TREK-1 can be linked to arrhythmia *via* mutations. See text for details.

To summarize our knowledge about the functional role of TREK-1 (Figure 1), it can be: 1/putative physiological roles are possible based on the electrophysiological properties of I_TREK-1_. 2/there are some changes in the expression of TREK-1 with some chronic disease that are involved in the development of the disease but the mechanisms are not understood. Different hypothesis can be put forward to explain this situation. The first is the lack of a selective blocker for TREK-1. Spadin is given as such a selective blocker, but its practical use is limited since the channel first has to be activated by arachidonic acid to be sensitive to spadin (Hivelin et al., 2016; Djillani et al., 2017; Djillani et al., 2019; Ma and Lewis, 2020). The second aspect is the pronounced species differences in K^+^ channel expression which provide an additional challenge for the elucidation of the role of TREK-1 in the heart. Finally, another aspect that has not been explored is the potential non-conducting properties of TREK-1. Indeed, in pathologies like breast cancer, such non-ionic properties of ion channels are well-known (Forzisi and Sesti, 2022). Whatever the explanation, it appears more and more obvious that TREK-1 plays a role in cardiac physiology and it represents a new target in cardiology.
